# Surgical Management of Pancreatic Head Arteriovenous Malformation: A Case Report

**DOI:** 10.7759/cureus.104054

**Published:** 2026-02-22

**Authors:** Anirudh Krishnan, Kellee Slater

**Affiliations:** 1 General Surgery, Princess Alexandra Hospital, Brisbane, AUS; 2 Hepatopancreaticobiliary Surgery, Princess Alexandra Hospital, Brisbane, AUS

**Keywords:** arterio venous malformations, benign biliary obstruction, hepatobiliary pancreatic surgery, inflammatory mass in the head of the pancreas, whipples operation

## Abstract

Pancreatic arteriovenous malformations (PAVMs) are uncommon abnormalities of pancreatic blood vessels that result in direct, high-flow connections between arteries and veins. There are currently fewer than 200 reported cases in the literature of this particular anomaly. They are often clinically asymptomatic but may present with abdominal pain, gastrointestinal bleeding, or features of portal hypertension, and can resemble cystic or neoplastic lesions on imaging. We report a case of a 48-year-old female with a pancreatic head mass presenting with non-specific abdominal pain and jaundice. Owing to diagnostic uncertainty and biliary obstruction, an open, stomach-preserving pancreaticoduodenectomy was performed. Intraoperatively, a tense blood-filled cyst was identified; histopathology demonstrated an organised haematoma with abnormal vascular channels consistent with a pancreatic arteriovenous malformation. This case highlights the diagnostic challenge posed by pancreatic arteriovenous malformations and supports surgical resection as definitive management when malignancy or obstruction cannot be confidently excluded.

## Introduction

Extrahepatic gastrointestinal (GI) arteriovenous malformations (AVMs) are a rare entity, most commonly diagnosed following presentation with upper or lower GI bleeding, and are most frequently identified in the caecum, ascending colon, and stomach. PAVMs represent a much smaller subset, with a reported prevalence of approximately 0.9% of all GI AVMs [1-3]. First described by Halpern et al. in 1968 in a patient with hereditary haemorrhagic telangiectasia (HHT), PAVMs consist of abnormal direct communications between arterial and venous systems without an intervening capillary bed [4].

This aberrant arteriovenous shunting results in high-flow haemodynamics, which may lead to venous congestion, portal hypertension, and local mass effect on adjacent structures. Consequently, PAVMs may produce symptoms that overlap with more common pancreatic pathologies, including obstructive jaundice, abdominal pain, and gastrointestinal bleeding. Although more than 90% of lesions are congenital, PAVMs may also occur secondary to trauma, pancreatitis, pancreatic transplantation, or within hypervascular tumours. While often asymptomatic, patients may present with vague or non-specific features such as weight loss, duodenal ulceration, or non-variceal upper GI bleeding [5].

This symptomatology, combined with non-specific radiologic findings, renders the diagnosis of PAVMs challenging and frequently results in delays to definitive management with transarterial embolisation (TAE) or surgical resection, including pancreaticoduodenectomy (Whipple’s procedure) or distal pancreatectomy. We present a rare case of a postoperative diagnosis of a PAVM treated with open resection as first-line therapy in a patient presenting with significant obstructive jaundice and abdominal pain.

## Case presentation

**Figure 1:**
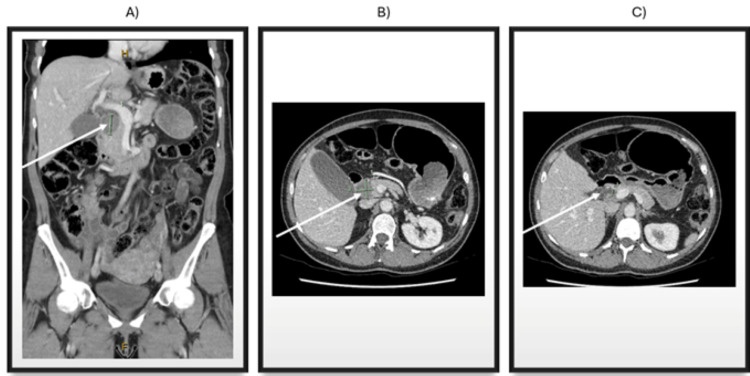
Abdominal portal venous contrast CT of cystic pancreatic lesion (white arrow) in A) coronal section demonstrating 20.5 mm tall, B) axial section demonstrating 26.2 mm long and 28.9 mm wide, with C) CBD dilated to 10 mm. CT: Computed tomography, CBD: Common bile duct

A 48-year-old Caucasian female was referred by her general practitioner to a private hepatobiliary surgeon for investigation of a new pancreatic head mass. This had been noted on an abdominal computer tomography (CT) scan (Figure 1) for acute-on-chronic epigastric and central abdominal pain, nausea, and loss of appetite. This was on a background of chronic constipation secondary to slow colonic transit, for which she was pending a colorectal surgical review in the coming weeks. The history of pain was not consistent with acute pancreatitis or cholecystitis/choledocholithiasis, and she had no history of significant alcohol use, smoking, or abuse of recreational drugs. She had no significant family history of pancreatic or GI malignancy. On abdominal examination, there was mild epigastric tenderness with a palpable gallbladder, but no other overt signs. Blood tests demonstrated liver function test derangement in an obstructive pattern, with a total bilirubin of 77 µmol/L, conjugated bilirubin of 52 µmol/L, ALP of 332 U/L, and GGT of 422 U/L. She had an otherwise normal complete blood count and normal tumour markers: Ca 19.9 and CEA.

**Figure 2:**
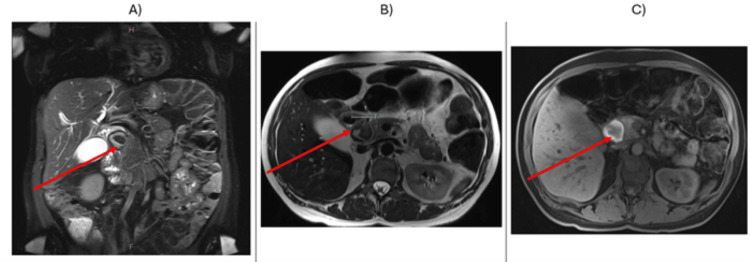
MR imaging of the biliary tract and pancreatic lesion (red arrow) in A) coronal T2 phase with evidence of biliary obstruction, B) axial T2 phase and C) T1 hyperintensity suggestive of protein-rich fluid within the cyst – highly suggestive of blood products.

A magnetic resonance cholangiopancreatography (MRCP) scan was undertaken, noting a 31 mm mass (Figure 2) in the pancreaticoduodenal groove with characteristics in keeping with a haematoma or proteinaceous cyst. An endoscopic ultrasound (EUS)-guided fine needle aspiration was conducted to sample the cystic contents. It showed low cyst fluid CEA levels and slightly elevated fluid amylase on biochemistry with blood, debris, and contaminant squamous cells on microscopy. There was no uptake within the lesion on a PET-DOTATATE and no evidence of thoracic metastases on a staging CT chest.

**Figure 3:**
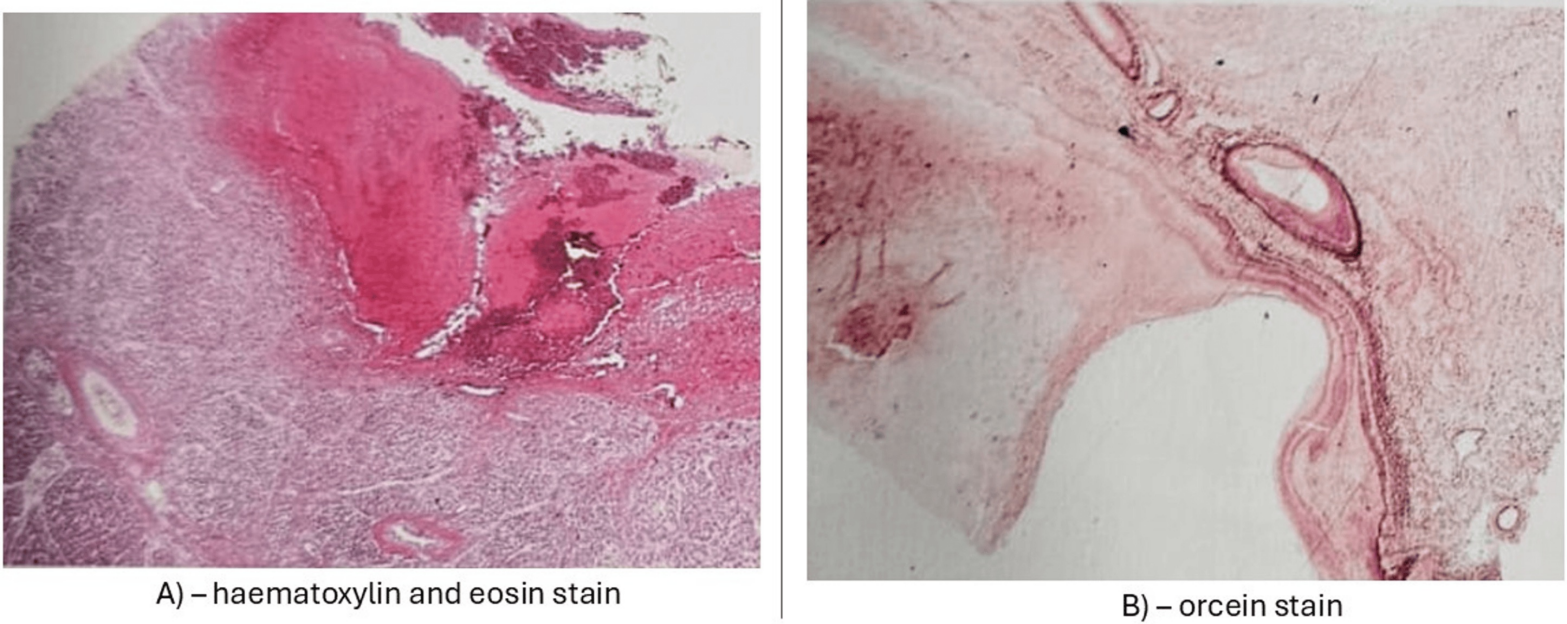
Two representative histopathologic slides of the resected specimen showing fibroblastic proliferation with haemosiderin deposition and minor inflammation (left: haematoxylin and eosin stain) with discontinuous layers of elastin fibres in the wall of the cavity (right: orcein stain).

The patient underwent an open conventional stomach-preserving pancreaticoduodenectomy (Whipple’s). The common bile duct (CBD) was dilated to 1 cm on transection, and the hepatic parenchyma exhibited changes of biliary congestion. There was no dilation of the pancreatic duct (PD) noted on division of the neck, and a retrocolic end-to-side pancreatojejunostomy was formed. Estimated blood loss was 500 ml. A 5 cm tense cystic lesion filled with old blood was noted in the head of the pancreas when opened on the back table. A minor incidental finding of a markedly redundant sigmoid colon, consistent with intermittent volvulus, was noted but did not require intervention. Her postoperative recovery was largely uncomplicated. On day three, a small volume of bile was noted in the surgical drains, in keeping with a low-grade bile leak. This was conservatively managed, and the drain was removed when there was no further output. The patient also had delayed gastric emptying requiring a brief period of total parenteral nutrition, which improved with prokinetic medication. She was discharged on day 14 from the hospital, and a follow-up CT showed expected post-operative changes without evidence of further biliary dilation or pancreatic lesions. The patient remains well with regular follow-up in an outpatient setting. Histopathologic examination of the resected specimen noted an organised haematoma with central old blood surrounded by fibroblastic proliferation and discontinuous elastin fibres in the wall of the cavity. Furthermore, there were irregular adjacent vessels with eccentric intimal thickening. These findings were most in keeping with a PAVM in the absence of trauma, significant inflammation, or malignancy (Figure 3).

## Discussion

Congenital PAVMs are most common (90%) and occur because of poor differentiation of primitive blood vessels, resulting in anomalous anastomoses between the arterial and portal venous networks, most often affecting the splenic artery, gastroduodenal artery, or small pancreatic arteries. They are noted in the head of the pancreas in 40-60% of cases, and only 10-30% are thought to be associated with HHT [6-8]. A handful of cases exist of PAVMs involving the entire body of the pancreas. Acquired PAVMs secondary to trauma or pancreatitis are frequently smaller, occurring due to pancreatic autodigestion and damage to pancreatic arteriolar and venular vessel walls, resulting in the formation of an AVM. Interestingly, secondary pancreatitis has been noted as a possible presentation in lesions in the tail of the pancreas, possibly owing to a clot or mass effect disrupting the PD [5]. Haemorrhagic manifestations are the commonest presentation with varying symptomatology depending on the location of the PAVM and its associated pathology. Upper GI bleeding is a common presentation secondary to haemorrhage into the CBD, PD, or duodenum due to compression against the affected organ and fistulation due to focal ischaemia [1,7]. Hakoda et al. describe an association with secondary portal hypertension in large PAVMs due to large-volume shunting of arterial blood directly into the portal venous system [9,10]. Thus, a further presentation of this condition can be complications of portal hypertension, such as oesophageal/gastric variceal bleeding or hepatosplenomegaly. The presentation in the above-described case was atypical: a cystic haematoma complicating a PAVM bleed with abdominal pain and obstructive jaundice. This demonstrates the significant complexity in distinguishing a causative aetiology given a wide range of presentations with several considerations when forming a management plan.

Pancreatic lesions such as cystadenocarcinomas, intraductal papillary mucinous neoplasms, serous cystadenomas, angiosarcomas, and neuroendocrine tumours can have standard CT appearances of a cystic or hypervascular lesion and should be excluded [9,11]. Angiography remains the definitive imaging modality to diagnose PAVMs and can demonstrate a “racemose” intrapancreatic vascular system, tortuous feeding arteries, and a dilated portal venous plexus appearing like a tangled nest of vessels [6,7]. However, this requires vascular catheterisation and may not be an appropriate investigation, especially if the pre-test probability is low. Abdominal Doppler sonography is an alternative imaging technique with characteristic features for PAVMs: a “mosaic” pattern of flow through the lesion and a pulsatile arterial waveform through the portal vein [5,12]. Multiphase or multiplanar CT can more accurately identify feeding vessels to the lesion compared to a single-phase CT with similar findings on T1- and T2-weighted scans [6]. A multiphase protocol CT and other angiographic studies were unfortunately not conducted in the described case, and contributed to the diagnostic difficulty. Finally, an EUS is a useful tool to interrogate pancreatic head lesions (where interventional gastroenterology is available) and can provide the option of histopathologic diagnosis as well, though the likelihood of an inadequate sample or exacerbating bleeding is high, as demonstrated in the above case [10]. Furthermore, the EUS conducted pre-operatively did not note the appearance of a vascular lesion on Doppler, possibly due to the presence of a haematoma impacting the reading of a "vascular thrill". Different classification systems exist for AVMs, which can be useful in identifying the most appropriate management in instances when the preoperative diagnosis is confirmed. Numerous studies suggest the system designed by Yakes et al. is most clinically applicable, utilising angiography to grade AVMs as types 1 to 4 depending on the number of arterial inputs, early/delayed venous filling, and microfistulae [13-15]. This system is among the most commonly used for categorising AVMs and for determining the risk of developing complications and the response to future management.

Surgical resection or TAE is the mainstay of definitive management of PAVMs, particularly in cases of obstructive jaundice, with limited evidence for radiotherapy. Some reports noted conservative monitoring of the lesion if asymptomatic and no portal hypertension [9]. TAE is a minimally invasive treatment involving the injection of coils, glue, polyvinyl alcohol particles, or covered stents into the feeding vessels of the lesion, most often accomplished with arterial catheterisation. The procedure carries a risk of duodenal/gastric ulceration, pancreatitis, and bowel ischaemia, especially if alcohol particles are used due to non-target embolisation [16]. Venous catheterisation is often not attempted due to the risk of thrombosis within the portal vein. Lesions classified as Yakes 3b or 4 were deemed more likely to be inadequately treated with TAE due to the number of feeding vessels and possibility for further collaterals post-treatment [16,17]. In cases of obstructive jaundice due to haematoma, TAE could reduce flow through feeding vessels but might not immediately improve the obstruction, potentially requiring an additional procedure such as CBD stenting. Wu et al. noted 38.5% of patients requiring resection post-TAE, with similar figures in the literature as the rate of recurrent bleeding post-embolisation [18]. Furthermore, it is well recognised that portal hypertension, once developed, may not improve following treatment or resection of the AVM, implying an element of time sensitivity [6,10]. The pathologic basis for this is thought to be related to the development of chronically arterialised portal flow resulting in obliterative portal venopathy and permanent alterations to hepatic vasculature [14,18]. Hence, timely surgical resection remains the gold standard treatment for this condition, though it is a more radical treatment with longer patient recovery times. The type of pancreatic resection and determination of laparoscopic, robotic, or open depends on the location of the lesion, its associated features (especially if there is portosystemic shunting), and the patient’s comorbidities. Intraoperative bleeding is an important consideration during mobilisation and handling of the pancreas, given the hypervascular structure, and many studies support the use of preoperative TAE to control flow into the lesion [16,19]. Another relevant aspect in our case, which highlights the practical reality, is that surgical resection is often pursued when a diagnosis is unclear and malignancy must be excluded. While TAE might have been considered had a PAVM been diagnosed pre-operatively, the presence of a cystic pancreatic head mass with obstructive jaundice justifiably prompted definitive surgery as a first-line treatment modality, both to diagnose and effectively treat the underlying pathology. Similarly, intraoperative frozen sections can be used to identify the pancreatic pathology, though it would rarely change the operative course.

## Conclusions

PAVMs are exceptionally rare and diagnostically challenging lesions that may closely mimic more common cystic or neoplastic pancreatic pathology. Their variable clinical presentation, ranging from incidental detection to haemorrhage, portal hypertension, or biliary obstruction, can delay recognition and significantly influence management decisions. In this case, several clinical and investigative features represented important diagnostic “red flags", including a blood-filled cystic pancreatic head lesion, obstructive jaundice in the absence of classic radiologic features of malignancy, and low cyst fluid tumour markers, all of which should prompt consideration of a vascular aetiology, as demonstrated by the EUS findings in the above case.

While advances in cross-sectional imaging and endoscopic ultrasound have improved lesion characterisation, definitive preoperative diagnosis of PAVMs remains challenging. Selective angiography, which was not performed in this case, may have demonstrated early venous filling and abnormal arteriovenous shunting, potentially facilitating preoperative diagnosis and consideration of alternative management strategies such as transarterial embolisation (TAE). However, in the setting of biliary obstruction, diagnostic uncertainty, and concern for malignancy, surgical resection remains the most definitive and durable treatment. This case underscores the importance of maintaining a broad differential diagnosis for pancreatic head masses, recognising key haemorrhagic and vascular imaging features, and individualising investigation and management based on lesion characteristics, symptom burden, and oncologic risk.
